# Autophagy, One of the Main Steps in Periodontitis Pathogenesis and Evolution

**DOI:** 10.3390/molecules25184338

**Published:** 2020-09-22

**Authors:** Maria Greabu, Francesca Giampieri, Marina Melescanu Imre, Maria Mohora, Alexandra Totan, Silviu Mirel Pituru, Ecaterina Ionescu

**Affiliations:** 1 Department of Biochemistry, Faculty of Dental Medicine, “Carol Davila” University of Medicine and Pharmacy, 020021 Bucharest, Romania; maria.greabu@umfcd.ro; 2 Department of Agricultural, Food and Environmental Sciences, Università Politecnica delle Marche, Via Ranieri 65, 60131 Ancona, Italy; f.giampieri@univpm.it; 3 Department of Complete Denture, Faculty of Dental Medicine, “Carol Davila” University of Medicine and Pharmacy, 020021 Bucharest, Romania; marina.imre@umfcd.ro; 4 Department of Biochemistry, Faculty of General Medicine, “Carol Davila” University of Medicine and Pharmacy, 020021 Bucharest, Romania; maria.mohora@umfcd.ro; 5 Department of Professional Organization and Medical Legislation-Malpractice, “Carol Davila” University of Medicine and Pharmacy, 020021 Bucharest, Romania; silviu.pituru@umfcd.ro; 6 Department of Orthodontics and Dento-Facial Orthopedics’, Faculty of Dental Medicine, “Carol Davila” University of Medicine and Pharmacy, 020021 Bucharest, Romania; ecaterina.ionescu@umfcd.ro

**Keywords:** periodontitis, autophagy, oxidative stress

## Abstract

Periodontitis represents a complex inflammatory disease that compromises the integrity of the tooth-supporting tissue through the interaction of specific periodontal pathogens and the host’s immune system. Experimental data help to outline the idea that the molecular way towards periodontitis initiation and progression presents four key steps: bacterial infection, inflammation, oxidative stress, and autophagy. The aim of this review is to outline the autophagy involvement in the pathogenesis and evolution of periodontitis from at least three points of view: periodontal pathogen invasion control, innate immune signaling pathways regulation and apoptosis inhibition in periodontal cells. The exact roles played by reactive oxygen species (ROS) inside the molecular mechanisms for autophagy initiation in periodontitis still require further investigation. However, clarifying the role and the mechanism of redox regulation of autophagy in the periodontitis context may be particularly beneficial for the elaboration of new therapeutic strategies.

## 1. Introduction

The tooth is an essential organ in humans. Teeth tissues have no (e.g., enamel) or very limited (e.g., dentine) regeneration capability because upon development completion only a limited number of stem cells persists in the mesenchyme (e.g., pulp and periodontal ligaments), and the epithelium disappears completely [1]. However, Bluteau et al. and Botelho et al. showed that stem cells are indeed important for tooth development and regeneration [1,2]. In addition to chondrocytes and osteoblasts, tooth pulp cells can trans-differentiate into other cell types such as neuron-like cells [1,2]. Therefore, maintenance of a healthy tooth is not only important for a fully functional digestive system but also essential to preserve an important cell source for regenerative medicine and stem cell therapies.

Although tooth diseases have a high prevalence [3], the linkage of the key cellular protection and cell death machinery (autophagy) regarding various dental diseases has not been sufficiently researched [4]. 

### 1.1. Periodontitis—Today’s Opinions and Tomorrow’s Perspectives

Oxidative stress (OS) and autophagy are considered closely inter-related, many key molecules being shared by the two processes [5,6]. However, the subtle interactions between ROS and autophagy in periodontitis remain unclarified [5,6]. Moreover, the exact mechanisms through which ROS are involved in autophagy initiation and regulation remain to be elucidated [5,6]. To contribute to the elucidation of this issue, this review focuses on the redox-sensitive pathways that lead to autophagy and summarizes the pathologic roles of OS and autophagy in a periodontal disease context [5,6].

Periodontitis is an inflammatory disease that specifically alters the integrity of the tooth-supporting tissue through a complex interaction between periodontal pathogens and the host’s immune response [7,8,9,10]. Traditional periodontal therapy is subgingival debridement and maintenance of good oral hygiene. Subgingival debridement is either the only treatment strategy or the initial phase before surgical intervention in severe periodontitis cases [7,8,9,10]. Mechanical therapy, represented by ultrasonic or hand instrumentation debridement, is the most common therapeutic strategy for the periodontal disease [7,8,9,10]. 

At present, the diagnosis and classification of periodontal diseases are almost entirely based on traditional clinical assessments [11]. Quantitative and qualitative analysis of the whole saliva (Table 1) and especially gingival crevicular fluid (Table 2) could provide potentially valuable additional information regarding the stage of periodontal disease. This information could be quite helpful in developing effective treatment strategies before placing the patients on a periodontal maintenance program [11].

A new approach of the periodontitis pathogenesis revealed that the pathogens alone are necessary but insufficient to initiate periodontal lesion development and progression. Periodontal tissue damage is caused mainly by undermining the host’s immune responses with the involvement of ROS [31,32].

The bacterial plaque-induced periodontal diseases are mixed infections that trigger an intense inflammatory reaction in the tissues around the teeth (site known as the periodontium) affecting its components [31,32].

Periodontal diseases can be divided into two general categories based on whether periodontal lesions are reversible or not [31,32]:

– Gingivitis is characterized by the presence of gingival inflammation without loss of gingival attachment. The affected site is limited to the gingiva [31,32].
– Periodontitis is an inflammatory disease of the periodontal tissues with a more complex pathogenic mechanism [31,32]. Periodontium plays essential supporting roles and includes the mineralized bonelike cementum, the periodontal ligament, and the alveolar bone. The periodontal ligament is a connective tissue between the cementum and the alveolar bone [33,34]. The main symptoms of periodontitis include apical migration of gingival attachment, the formation of a periodontal pocket, and progressive periodontal bone loss. Periodontitis progression culminates with tooth loss [11,35].

Nowadays, due to their high prevalence, periodontal diseases should be regarded as a real and serious health problem in our society [36]. Gingivitis prevalence is over 80%, a peak being recorded during the pubertal period [36]. Chronic periodontitis affects about 50% of the European population with 10% of the affected people suffering from an aggressive form. At 60–65 years of age, the prevalence can increase up to 70–85% [36]. Presently, periodontal diseases should be considered as one of the most common ailments. 

Many key questions regarding the molecular mechanisms of periodontitis pathogenic processes remain unanswered. For instance, it is still unclear why, in some cases, bacterial infection-induced tissue damages are limited to the gingiva, while in other cases it evolves toward alveolar bone loss [36]. At the same time, the degree of bone resorption could be more or less severe [36]. To explain these clinical observations, research has been focused on the molecular particularities of the host’s response to the microbial attack.

Experimental data help to outline the idea that the molecular way towards periodontitis initiation and progression presents four key steps: (1) bacterial infection; (2) inflammation; (3) oxidative stress; and (4) autophagy.

### 1.2. Periodontitis Initiation and Progression Steps

#### 1.2.1. Step 1: Bacterial Infection

Traditionally speaking, the main cause of periodontal diseases is represented by specific bacterial species that colonize subgingival sites or pockets [11,17,18,36]. These bacteria are responsible for the inflammatory reaction of various intensities [11,17,18,36]. Currently, specific treatment still consists of reducing and eliminating subgingival microorganisms [11,17,18,36]. However, there are some significant limitations of the current periodontitis treatment strategies: they are not successful in all the patients; there are no available protocols to identify the susceptible population groups; prevention is not feasible; and the complete regeneration strategies of periodontal affected tissues is still impracticable [36].

Clinical evidence shows that bacterial plaque is not the main contributor in some aggressive forms of periodontitis [11,36]. In such cases, the control of periodontal diseases becomes even more difficult [11]. There are certain microorganisms related to periodontal disease’s pathogenesis; however, according to Mendes et al., the experimental evidence does not support the periodontal pathogen invasion as the only key step in this process [37]. Virulent oral microbiota presence must be considered as one of the first steps toward periodontitis [38]. Accumulation of these bacteria is a starting point for affecting the supporting structures’ integrity, followed by the alveolar bone loss [38]. In other words, periodontitis pathogenesis is based on the imbalance between microbiota/dental biofilm and the host inflammatory response. Dysbiosis is regarded as a consequence of oral and gut microbiota imbalance with long term consequences [39]. Inchingolo et al. recognized that oral dysbiosis triggers a highly inflammatory condition, and it should be considered an underestimated chronic periodontitis key factor, along with altered pro- and anti-inflammatory gene responses [40]. As a result, Inchingolo et al. conducted a study focused on attempting periodontitis and dental caries prevention with the help of various probiotics [41]. Ballini et al. highlighted the key role of bone host cell invasion by *Porphyromonas gingivalis* in the pathogenesis of bone disorders, as well as interesting scientific evidence sustaining cytokines’ important roles in the molecular mechanism of bone disease [42]. Recent experimental evidence strongly sustains the idea that the host’s inflammatory response represents the main factor affecting the stage of periodontitis [36,43].

#### 1.2.2. Step 2: Inflammation

Inflammation should be regarded as the common denominator between periodontitis and systemic diseases such as metabolic syndrome, diabetes, and atherosclerosis [7,8,36]. These systemic diseases, together with periodontitis, have a complex etiology based on a puzzle of changeable (age or genetic predisposition) and modifiable risk factors (smoking, alcohol, dyslipidemia, and chronic infections) [39]. Inflammation represents a constellation of physiological responses to stress and involves many complex processes: aggression recognition, inflammasome activation, energy supply triggering the alteration of the cellular redox homeostasis followed by aggression elimination via autophagy or apoptosis, and recovery [36].

Clinical evidence reveals that chronic inflammatory reactions usually lead to the progressive damage of cells in the periodontium [8]. Commonly, proper inflammation responses are beneficial for the host defense; however, excessive inflammatory reactions can lead to serious damage, tissue destruction, or organ failure [8].

Periodontal uncontrolled inflammation reactions are mainly caused by complex microbial communities’ transition from a commensal to a pathogenic entity [40]. Biochemical communication among constituent bacterial species generates a polymicrobial synergy between metabolically compatible entities. The keystone pathogens can enhance the constituent community virulence [40]. The resulting dysbiotic community will impair specific host immunity aspects to further decrease immune surveillance while initiating an overall inflammatory response [40]. Moreover, inflammophilic organisms depend on protein residues derived from inflammatory tissue breakdown [40]. Dysbiosis and inflammation amplify each other, and the resulting environmental changes will further affect the pathological bacterial community [40]. 

#### 1.2.3. Step 3: Oxidative Stress

Keystone or keystone-like periodontitis pathogens, predominantly Gram-negative anaerobic or microaerophilic bacteria, are appreciably sensitive to changes in the redox equilibrium [40]. Consequently, ROS can act as a chemical weapon against the keystone pathogens. Experimental evidence shows a marked increase in ROS formation when leukocytes were treated with *Porphyromonas gingivalis (P. gingivalis)* lipopolysaccharide or *Fusobacterium nucleatum* (*F. nucleatum*), in vitro [44,45,46]. In addition, a clinical study reported that ROS serum levels are positively correlated with immunoglobulin G antibodies to specific periodontal pathogens: *P. gingivalis, Aggregatibacter actinomycetemcomitans*, and *Prevotella intermedia* [47].

It is important to emphasize that cellular ROS, at basal levels, are extremely important for eukaryotic cell physiologic processes: cellular signaling, signals transduction, cellular differentiation, apoptosis, and autophagy [48,49]. H_2_O_2_, for instance, is one of the main factors in redox-sensitive signaling pathways [50]. Choe et al. showed that H_2_O_2_, continuously formed by glucose oxidase activity, in low concentrations, induces periodontal ligament fibroblasts proliferation and osteoblastic differentiation via the runt-related transcription factor-2 (Runx2) [50]. It also has been shown that in periodontal tissues H_2_O_2_ could initiate defensive inflammatory responses by activating mitogen protein kinase (MAPK) and NF-kB [51]. Moreover, Cavalla et al. showed that H_2_O_2_ could enhance the gelatinolytic matrix metalloproteinases (MMPs) activity, stimulating the MMP-dependent migration of the periodontal ligament fibroblasts [52]. These experimental findings suggest that H_2_O_2_, along with other ROS, is involved in periodontal ligament fibroblasts’ proliferation and differentiation. However, cellular responses to H_2_O_2_ may differ depending on concentration and cell type [53]. According to Burdon et al., 1 μM of H_2_O_2_ promoted the proliferation of BHK-21 fibroblasts, while 0.5 and 1 mM H_2_O_2_ induced cell death via apoptosis [53]. ROS has multifaceted effects. Significant experimental evidence shows that, in living systems, ROS and nitrogen species act as a double-edged sword because they can serve as molecular signals, initiating protective stress responses beneficial to the organism, but are able to cause oxidative damage and cellular dysfunction [49].

Normally, to maintain redox homeostasis, ROS generation occurs in equilibrium with the release of a complex group of ROS scavengers: glutaredoxin and thioredoxin systems, enzymes (SOD, GPX, and CAT), and small molecules (vitamin C and reduced glutathione (GSH)) [54,55]. The redox balance between the generation and inactivation of ROS is extremely important for human health [17]. Excessive ROS production decreases antioxidant levels. Inhibition of antioxidant enzymes are the main cause that paves the way to OS installation [17]. OS may induce indiscriminate damage to biological macromolecules (lipids, proteins, and DNA) [49]. The homeostatic imbalance between the antioxidant defense systems and ROS leads to oxidative responses believed to be the main initiators of periodontal damage [56,57].

In the context of periodontitis, periodontal tissue damage may be caused directly by the installed OS and indirectly via the activation of cell signaling pathways involved in inflammation, autophagy, and apoptosis [17,18].

Experimental evidence reveals that direct ROS induced periodontal tissue damage could be mediated through: (1) mitochondrial injury and ROS bursts [58,59]; (2) lipid peroxidation and cell membrane destruction [60]; (3) protein oxidative damage and denaturation; (4) enzyme inhibition [18,23,25,61]; and (5) nucleic acid damage (strand breaks and base pair mutations) [12]. A more complex question arises: How does ROS induce periodontal tissue damage via regulating signal transduction and gene transcription? In this regard, four possible pathways have been described:

1. ROS are able to activate NF-kB, initiating a signaling cascade involved in inflammatory and immune responses [62].
2. ROS can activate JNK, initiating cell apoptosis [63].
3. ROS are involved in inflammasome formation and activation, triggering cell death [64].
4. ROS are the main contributors in the autophagy stage [65].

Next, we focus on the mechanisms of ROS-mediated activation of autophagy in the context of periodontitis. 

#### 1.2.4. Step 4: Autophagy

Autophagy is an evolutionarily conserved intracellular degradation system, designed to deliver damaged cytoplasmic material (damaged organelles, denatured proteins, and bacteria) to the lysosome and to recycle the degradation products for the anabolic pathways and/or energy production [65,66]. 

Four different forms of autophagy are known: micro-autophagy, chaperone-mediated autophagy, macro-autophagy, and non-canonical autophagy. Among these forms, macro-autophagy (further referred to as autophagy) is the most widely investigated type [4,5,6,67]. Following the pathway of autophagy, cells are able to coordinate energy and biomacromolecules precursors needed for important cellular processes (e.g., growth and proliferation) with the extracellular stimuli and carbon source (such as amino acids and glucose) availability [4,5,6,67]. If energy and/or exogenous carbon sources are not sufficient to maintain the rate of protein synthesis or to sustain the metabolic reactions, then cells will initiate autophagy in order to rapidly degrade the old or burned-out molecules and reuse the new-generated pool of precursors [4,5,6,67].

Unlike other intracellular degradation pathways, autophagy characteristically sequesters intracellular material inside a double-membrane vesicle named autophagosome. After its formation, the autophagosome fuses with lysosomes, triggering the double-membrane vesicle degradation [68]. The complete autophagy pathway follows five highly regulated steps: induction, elongation, maturation, transport to lysosomes, and degradation [68].

### 1.3. Autophagy in Periodontitis Context

#### 1.3.1. Oxidative Stress and Autophagy: A Double Sense Connection 

An important issue is the way by which the autophagic machinery pathway can be intersected by OS. Experimental data have highlighted that antioxidant treatment can prevent autophagy, suggesting that the redox imbalance is a main player in this degradative process [69,70,71,72,73].

The very fast autophagy induction upon mitochondrial ROS production sustains a rapid switch response, mediated by redox-sensitive proteins, among which AMPK could be the main contributor. It has been proposed that AMPK could be activated upon H_2_O_2_ exposure, most probably through *S*-glutathionylation of reactive cysteines located at the *α*-(Cys^299^ and Cys^304^) and *β*-subunits (still unidentified) [70,71]. Recent studies have shown that in nutrient deprivation, cell actively extrudes GSH using the drug efflux pump, multidrug resistance protein 1 (MRP1), to make the intracellular redox environment more oxidizing. These redox changes will trigger oxidative modifications of the prime redox-sensitive proteins [69]. Evidence that GSH chemically induced oxidation can initiate autophagy, even in the absence of any autophagic stimulus, highlights the thiol redox homeostasis’ importance in the complex molecular mechanism of autophagy [69]. This idea is also sustained by experimental evidence indicating that the biological functions of many proteins involved in both induction and progression are based on Cys residues. In this regard, the following proteins should be mentioned: the tensin homolog (PTEN), the two ubiquitin-like systems Atg7–Atg3 and Atg7–Atg10, and some members of Rab GTPase [72]. p62 contains a Cys-rich zinc-finger motif essential for metal binding and is susceptible to redox regulation. Despite the absence of experimental data proving the possible redox sensitivity of p62, it could be speculated that similar to other ZZ-containing proteins, p62 could also undergo oxidative induced structural alterations able to influence its role on the autophagy stage [72].

He et al. revealed that increased levels of ROS can activate NF-kB, triggering the upregulation of Beclin1 [74]. Moreover, the JNK signaling activation during OS leads to the phosphorylation of Bcl-2, which induces Beclin 1 dissociation from the Vps34 complex and, consequently, activates autophagy [75]. Several studies have outlined the relevance of Beclin 1 in periodontitis. Specifically, An et al. reported higher protein expression levels of LC3II/I and Beclin 1, as well as enhanced transcriptional levels of Atg7, Atg12, Beclin-1, and LC3 in periodontal ligament stem cells isolated from patients with periodontitis compared with healthy individuals [76].

In addition, ROS can induce autophagy by activating the Atg12–Atg5 complex [52]. The Atg12–Atg5 conjugate is a ubiquitin-like protein complex that is essential for autophagophore elongation in autophagy [11,12]. Mai et al. revealed evidence of the delicate adjustment of Atg12–Atg5 depending on the intracellular redox state [77]. 

**Figure 1:**
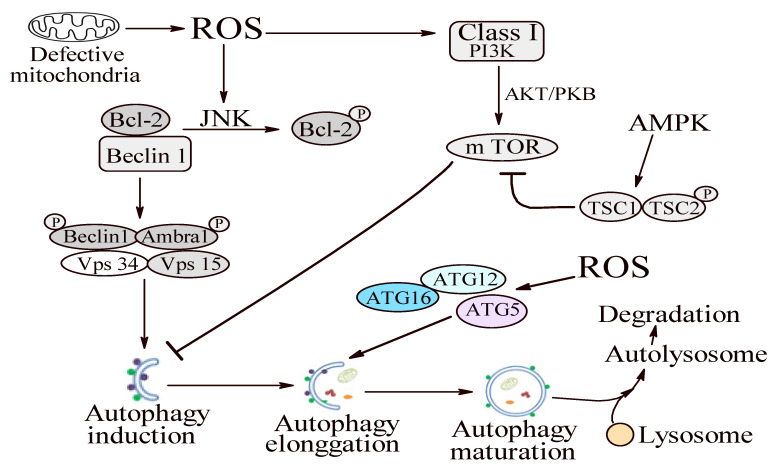
Schematic representation of the possible mechanism of autophagy regulation in the periodontitis context. Autophagy can be modulated by ROS via four different pathways: (1) Atg12–Atg5 complex activation, promoting autophagy elongation; (2) ROS-dependent JNK induced Bcl-2 phosphorylation triggering Beclin 1 dissociation and autophagy induction; (3) PI3K-AKT pathway initiation triggering the activation of mTOR, which, in turn, acts as an autophagy induction inhibitor; and (4) the AMPK-dependent TORC1 activity inhibition leading to autophagy activation. Adapted from Liu C. et al. [51].

Mitochondrial ROS have been identified as important signaling molecules in regulating autophagy [51]. Elevated ROS levels can adjust autophagy development by controlling autophagy-related genes (Atgs) and/or the upstream signaling pathways via targeting mammalian target of rapamycin complex 1 (mTORC1), Beclin 1, and the Atg12–Atg5 complex (Figure 1) [51]. 

Autophagy can also play an important role in mitochondrial ROS generation and scavenging. Furthermore, Bullon et al. showed that decreased mitochondrial ROS production induced a slowdown of autophagy [78].

Many signals, such as rapamycin, insulin, and OS, regulate mTORC1 activity. Studies are revealing that ROS can influence mTORC1 activity through the tuberous sclerosis complex 1/2 (TSC1/TSC2) heterodimer (Figure 1) [79,80,81,82]. Increased levels of ROS activate AMP-activated protein kinase (AMPK). Activated AMPK induces TSC2 phosphorylation and TSC1/TSC2 complex activation, thus inhibiting mTORC1 and stimulating ULK (an important initiator of the autophagy complex) to induce autophagy (Figure 1) [80,81,82]. Conversely, ROS can activate the phosphoinositide-3-kinase (PI3K)-protein kinase B (Akt)-mTORC1 signaling pathway by directly activating PI3K or by controlling the Akt phosphorylation state, therefore inhibiting autophagy induction (Figure 1) [81,82,83]. Stafford et al. revealed that *P. gingivalis* invasion induced the mTOR pathway inhibition in oral epithelial cells, representing the first reported evidence that conferred a potential role to mTORC1 in the molecular landscape of periodontitis [84,85].

Briefly, the data presented above lead to the conclusion that the possible autophagy ROS-dependent modulation is based on at least four mechanisms: (1) the Atg12–Atg5 complex activation, promoting autophagy elongation; (2) ROS-dependent JNK induced Bcl-2 phosphorylation triggering Beclin 1 dissociation and autophagy induction; (3) PI3K-AKT pathway initiation triggering the activation of mTOR, which, in turn, acts as an autophagy induction inhibitor; and (4) AMPK-dependent TORC1 activity inhibition leading to autophagy activation (Figure 1) [51]. 

Autophagy can also play an important role in mitochondrial ROS generation and scavenging, predominantly by the Nrf2 release and activation [86]. Interesting evidence has indicated that Nrf2 and its target genes are extremely important for cellular redox homeostasis maintenance in order to limit the OS-associated periodontal damage [47,85,87,88].

#### 1.3.2. Autophagy—A Possible Antioxidant Pathway?

Filomeni et al. showed that antioxidant response and autophagy are mechanisms simultaneously induced by OS to concomitantly decrease ROS concentration and reduce biomolecules oxidative damage [65]. This complex orchestrated repair system perfectly fits the needs of a cell trying to find a new homeostatic state. 

All data available so far sustain the fact that autophagy responds very rapidly to OS and plays a crucial role in decreasing the toxicity of oxidized and damaged molecules through their selective removal [65]. Consequently, autophagy should be included, in principle, in the large family of antioxidant processes. 

#### 1.3.3. Autophagy—A Janus God in Periodontitis

In previous studies, depending on the context, the induction of autophagy has been shown to have both protective and pathological effects in periodontitis [65].

Autophagy involvement in the pathogenesis and evolution of periodontitis should be regarded from at least three points of view: (1) periodontal pathogen invasion control; (2) innate immune signaling pathways regulation; and (3) apoptosis inhibition in periodontal cells.

##### Periodontal Pathogen Invasion Control by Autophagy 

Numerous studies have highlighted the strong association of periodontitis with microbial infection. Antimicrobial autophagy represents a sequential set of molecular barriers against incoming bacteria [89,90]. To unleash the autophagy machinery, mammalian cells have guiding systems for detecting the location, intensity, and extent of the pathogen attack [89,90]. These guiding systems include the pattern recognition receptors (PRRs), active at any stage, and the autophagic adaptors, which become active if bacteria penetrate the cytosol, and orchestrate the bacterial elimination [89,90]. PRRs can induce autophagic responses at different stages of host–bacteria encounters [89,90]. Thus, autophagy initiation can occur before, during adhesion and pathogen-induced uptake by the host cell, or during phagocytosis of the bacteria cell in macrophages [89]. The autophagic adaptors are represented by Sequestosome 1/p62-like receptors (SLRs) [78]. SLRs recognize molecular signals (ubiquitin, galectin, and membrane phospholipid modifications), expressed on damaged host membranes associated with the microbe or on the invading bacteria, and further physically recruit and organize the autophagic machinery [89,90].

Regarded as an intracellular innate defense pathway, autophagy is usually enhanced in infected cells, being deeply involved in the cellular antimicrobial defense mechanisms [91]. For instance, Kim et al. reported that autophagy can inactivate and eliminate intracellular pathogens such as *Mycobacterium tuberculosis* (*M. tuberculosis*) [89]. However, to avoid lysosomal degradation, many pathogens, including *Legionellapneumophila* (*L. pneumophila*), have developed autophagy suppressing strategies [51]. The microbial countermeasures for undermining host cell autophagy mechanisms often involve: (1) Beclin 1 targeting to prevent autophagosomal maturation, block autophagy, or activate autophagy to produce nutrients for microbes; (2) autophagosomal membrane perforation to prevent acidification; (3) mAtg8s cleavage; and (4) SLRs recognition epitopes masking [89]. 

Interestingly, in vitro studies conducted on cultured cells exposed to bacterial species have suggested that periodontal pathogens such as *P. gingivalis* could induce autophagy [91]. Belanger et al. revealed that, in human coronary artery endothelial cells, *P. gingivalis* was able to traffic rapidly from phagosomes to autophagosomes [91]. These results are also sustained by the finding that ROS, generated in the presence of *P. gingivalis*, induced the LC3 levels to increase [92]. Macrophages exposure to *P. gingivalis* led to increased autophagosomes and autophagolysosomes assembly [92]. Park et al. showed that *P gingivalis* activated LC3-I/LC3-II conversion and increased the conjugation of autophagy-related ATG5–ATG12 and Beclin 1 expression [92]. The expressions of Beclin 1, ATG5–ATG12 conjugate, and LC3-II were significantly inhibited by the autophagy inhibitor, methyladenine. Interestingly, methyladenine increased *P. gingivalis* survival and the proinflammatory cytokine, interleukin-1β, production [92]. In turn, macrophages can eliminate *P. gingivalis* via an autophagic response that triggers the limitation of an excessive inflammatory response by downregulating interleukin-1β production [92]. All of these results outline the conclusion that autophagy induction by *P. gingivalis* may play a main role in the inflammatory reactions scene in the context of periodontitis. 

In addition, taken together, these results strongly suggest that autophagy induction can sustain specific periodontal bacterial species survival by replication within an autophagosome-like compartment [51]. 

Interestingly, no LC3 level increase was found in *A. actinomycetemcomitans* infected cells [93]. Blasi et al. suggested that the activity of cellular autophagy involvement in the complex cellular response to an infection is associated, probably, only with periodontal bacterial species [93]. 

##### Autophagy and Innate Immune Signaling Pathway Regulation 

Autophagy is initiated by pattern recognition receptors and autophagic adaptors and has important effects on immunity [89,90]. Autophagy poses its own set of PRRs, the SLR adaptors, to eliminate invading microbes [89,90,94]. As mentioned above, some bacterial species have developed molecular strategies to avoid autophagic destruction [89,90,94]. During evolution, almost all innate immunity systems, including inflammasome components and the conventional PRRs, have become more interrelated with the autophagy pathway [89,90,94]. Autophagy acts as a modulator of classical PRRs, such as Toll-like receptors (TLRs) and Nod-like receptors (NLRs) [89,94]. 

The inflammation mechanism is controlled by autophagy via regulatory interactions with the innate immune signaling pathways [89,90,94]. The autophagic machinery can remove endogenous inflammasome agonists and control immune mediators’ secretion [89,90,94]. Moreover, autophagy contributes to antigen presentation and T cells, influencing their further behavior [89]. Consequently, it can be presumed that autophagy may control the periodontal innate immune response in two possible manners:

- the inflammasome-dependent manner [51]; and
- the inflammasome-independent way [51].

a. The Inflammasome-Dependent Manner

The inflammasome, a real signaling platform, consists of a group of cytosolic innate immunity complexes assembled in response to pathogen-associated molecular patterns (PAMPs) and endogenous alarmins/danger-associated molecular patterns (DAMPs) [95]. An inflammasome consists of pro-caspase 1, ASC adaptor, and one of the sensor proteins from the NLR family or the PYHIN family [89]. Briefly, inflammasomes can be divided into two families: the NOD-like receptor (NLR) family and the pyrin and HIN200 (hematopoietic interferon-inducible nuclear antigens with 200 amino-acid repeats) domain-containing protein (PYHIN) family [95]. Each inflammasome type is induced as a response to numerous different exogenous and endogenous signals [96]. After PAMP/DAMP-induced assembly, the inflammasome will respond by initiating the caspase 1-dependent pro-IL-1β and pro-IL-18 cytosolic processing into the mature forms (IL-1β and IL-18, respectively) and their secretion [89,97]. 

Autophagy has a negative role in inflammasome activation [51]. Animal studies have shown that mice lacking LC3B (a ubiquitin-like protein important for autophagosome formation and maturation) produced higher levels of caspase-1-dependent cytokines than wild-type mice [51,98]. Under sterile conditions, autophagy’s mission is cytoplasm cleaning of debris—aggregates that can act as endogenous inflammasome agonists [89].

As a matter of fact, the functional recognition of autophagy as an anti-inflammatory player arises from the findings illustrating increased IL-1β and IL-18 production and associated intestinal inflammation in a mouse model of CD with defective Atg16L1 (an evolutionarily conserved protein, playing a key role in autophagy pathway) [99].

Moreover, autophagy may be involved in unconventional secretion of cytosolic proteins with extracellular immune functions [89,90]. The exact molecular mechanism of how this occurs is still unclear. IL-1β and IL-18 do not have signal peptides for entering the ER and following the conventional secretory pathway (ER–Golgi–plasma membrane) [89,90]. Instead, they can be delivered to the extracellular environment via an unconventional secretion pathway [89]. Although autophagy suppresses inflammasome assembly and activation under normal (nutrient-rich) conditions, autophagy can contribute to the unconventional secretion of IL-1β under stress signals, e.g., infection [89]. These molecular events could be regarded as parts of a possible double-lock mechanism meant to repress inflammasome activity under basal conditions, but to increase it temporarily in response to infection [89]. Autophagy can suppress the inflammasome basal level of activity by continuously removing [100,101] the endogenous sources of inflammasome agonists such as ROS and mitochondrial DNA [100,101].

b. The Inflammasome-Independent Manner

All data reported thus far [100,101,102,103] sustain the conclusion that autophagy acts as a negative controller of inflammasome activation. 

Interestingly, it has been highlighted that autophagy negatively regulates the secretion of IL-1α [104]. Castillo et al. found that autophagy simultaneously plays a dual role against tuberculosis: antibacterial and anti-inflammatory. *M. tuberculosis* infection of mice lacking Atg5 resulted in increased bacillary burden and excessive pulmonary inflammation characterized by neutrophil infiltration and increased IL-1α levels [104]. Thus, Castillo et al. concluded that autophagy has double protective action in vivo by suppressing *M. tuberculosis* growth and the potential damaging inflammation response [104].

All of these presented findings lead to the conclusion that autophagy might influence periodontal inflammatory response by regulating inflammation in two ways: inflammasome-dependent and inflammasome-independent. 

In humans, the failure of key components of the autophagic apparatus can lead to chronic inflammatory, autoimmune, or general immunity disorders. Actual knowledge regarding the immunological roles of autophagy is still in its infancy and many interesting puzzles and important questions remain to be resolved.

##### Periodontal Cells Protection against Apoptosis 

Bullon et al. showed the inhibition of autophagy in gingival fibroblasts treated with *P. gingivalis* lipopolysaccharide-induced apoptosis. Their results suggest a possible protective role of autophagy [78]. 

An et al. revealed enhanced LC3 expression and autophagosome generation in inflamed periodontal ligament tissues [75].

However, the molecular mechanism underlying the protective role of autophagy remains hidden for the time being. A new hypothesis in this regard could be the blocking of autophagy which would trigger apoptosis initiation. 

## 2. Conclusions

The progress in the molecular landscape of autophagy redox regulation has provided interesting details regarding the connection mechanisms between ROS and autophagy. Scientific evidence highlights ROS roles as upstream autophagy modulators. In turn, cellular redox status can be regulated by autophagy via the Nrf2 signaling pathway. 

Numerous studies highlighted the key roles of ROS in periodontitis molecular mechanisms. Furthermore, recent data are increasingly outlining the subtle but important role played by autophagy in periodontitis pathogenesis. Moreover, autophagy and ROS molecular connections, in the periodontitis context, are increasingly attracting the attention of scientists. Starting from the accumulated scientific data, we may speculate that the redox regulation of autophagy is a serious candidate for one of the main roles in periodontitis pathogenesis. 

Experimental data lead to the conclusion that, in the context of periodontitis, excessive ROS generation triggers intensive inflammatory reactions, apoptosis, and disturbs autophagy activity, inducing periodontal tissue alterations. Conversely, it is believed that autophagy redox regulation is an efficient and effective mechanism for antibacterial responses and can be associated with periodontal cell protection against apoptosis. 

However, the exact roles played by ROS inside the molecular mechanisms for autophagy initiation and progression in periodontitis still require further investigation. In addition, regarding autophagy involvement in periodontitis pathogenesis and progression, there are still many questions to be answered. 

Nevertheless, clarifying the role and the mechanism of redox regulation of autophagy in the context of periodontitis may be particularly useful to pave the way towards new and more efficient therapeutic strategies. 

This will be considered in future work.

## Figures and Tables

**Table 1:** Levels of some salivary parameters as possible future periodontitis biomarkers in periodontitis patients compared with controls.

Marker	Type of Marker	Pattern
8-hydroxy-2-deoxyguanosine (8-OHdG)	Oxidative stress DNA damage marker	Increased in saliva [12,13,14,15,16,17,18] No significant change in saliva [13]
Malondialdehyde (MDA)	Oxidative stress protein damage marker	Increased in saliva [17,18,19,20,21,22,23]
Protein carbonylation	Oxidative stress protein damage marker	Increased in saliva [15,18]
Salivary total antioxidant capacity	Antioxidant	Decreased in saliva [17]
Uric acid	Antioxidant	Decreased in saliva [17]
Reduced and oxidized glutathione (GSH and GSSG)	Antioxidant	Decreased in saliva [24]
Superoxide dismutase (SOD)	Enzymatic antioxidant	Decreased in saliva [23,25] Increased in saliva [26]
Glutathione peroxidase (GPX)	Enzymatic antioxidant	Decreased in saliva [17,23] No significant change in saliva [24]
matrix metalloproteinases-8	Bone loss marker	Increased in saliva [17,27]
C-terminal telopeptide of type I collagen (CTX I)	Bone loss marker	Increased in saliva [17]
IL-1β	Cytokines	Increased [28,29]
IL-6	Cytokines	Increased [28,29]
IFN-γ	Cytokines	Increased [28,29]

**Table 2:** Levels of some GCF parameters as possible future periodontitis biomarkers in periodontitis patients compared with controls.

Marker	Type of Marker	Pattern
Malondialdehyde (MDA)	Oxidative stress protein damage marker	Increased [20,26]
8-hydroxy-2-deoxyguanosine (8-OHdG)	Oxidative stress DNA damage marker	Increased [30]
Superoxide dismutase (SOD)	Enzymatic antioxidant	Increased [26]
Reduced and oxidized glutathione (GSH and GSSG)	Antioxidant	Decreased [31]
IL-1β	Cytokines	Increased [28,29]
IL-6	Cytokines	Increased [28,29]
IFN-γ	Cytokines	Increased [28,29]
